# A Case of Asymmetric Nipple Enhancement as an Imaging Precursor to Invasive Ductal Carcinoma

**DOI:** 10.7759/cureus.9514

**Published:** 2020-08-01

**Authors:** Swati Sharma, Erick Blaudeau, Smita Sharma

**Affiliations:** 1 Radiology, University of Florida College of Medicine, Jacksonville, USA

**Keywords:** breast cancer, nipple enhancement, abnormal ct

## Abstract

On multidetector computed tomography (CT), it is important to scrutinize the imaged portions of the breasts. In recent years, the dramatic rise in CT imaging has led to the increased detection of incidental breast lesions. We describe a case of invasive ductal carcinoma that presented as stage IV cancer, and retrospective review of prior imaging study revealed asymmetric nipple enhancement on a trauma protocol CT chest acquired three years earlier. This report highlights the importance of being attentive to breast abnormalities on CT performed for indications other than breast disease and additionally focuses on the approach to address abnormal enhancement of the nipple areolar complex (NAC).

## Introduction

Mammography is currently the preferred examination for breast cancer screening; however, computed tomography (CT) often provides initial imaging of the breast when scanning is performed for other indications. Breast abnormalities can be overlooked at CT or inaccurately assessed, despite the ability of CT to reveal sufficient detail. Radiologists should carefully evaluate the imaged portions of the breasts and adjacent soft tissues on every chest or abdominal CT [1,2]. Approximately 8% of breast cancers arise in the region of the central mammary ducts near the nipple [3]. Abnormal nipple enhancement identified on MRI and recommendations to address it have been discussed in the literature. By sharing this case of invasive ductal carcinoma that initially presented as asymmetric nipple enhancement, we will also review the anatomy of the nipple areolar complex (NAC) and discuss an approach to further workup abnormal enhancement of NAC.

## Case presentation

We report an unusual case of incidental asymmetric nipple enhancement that later developed into a fungating breast mass. 

**Figure 1:**
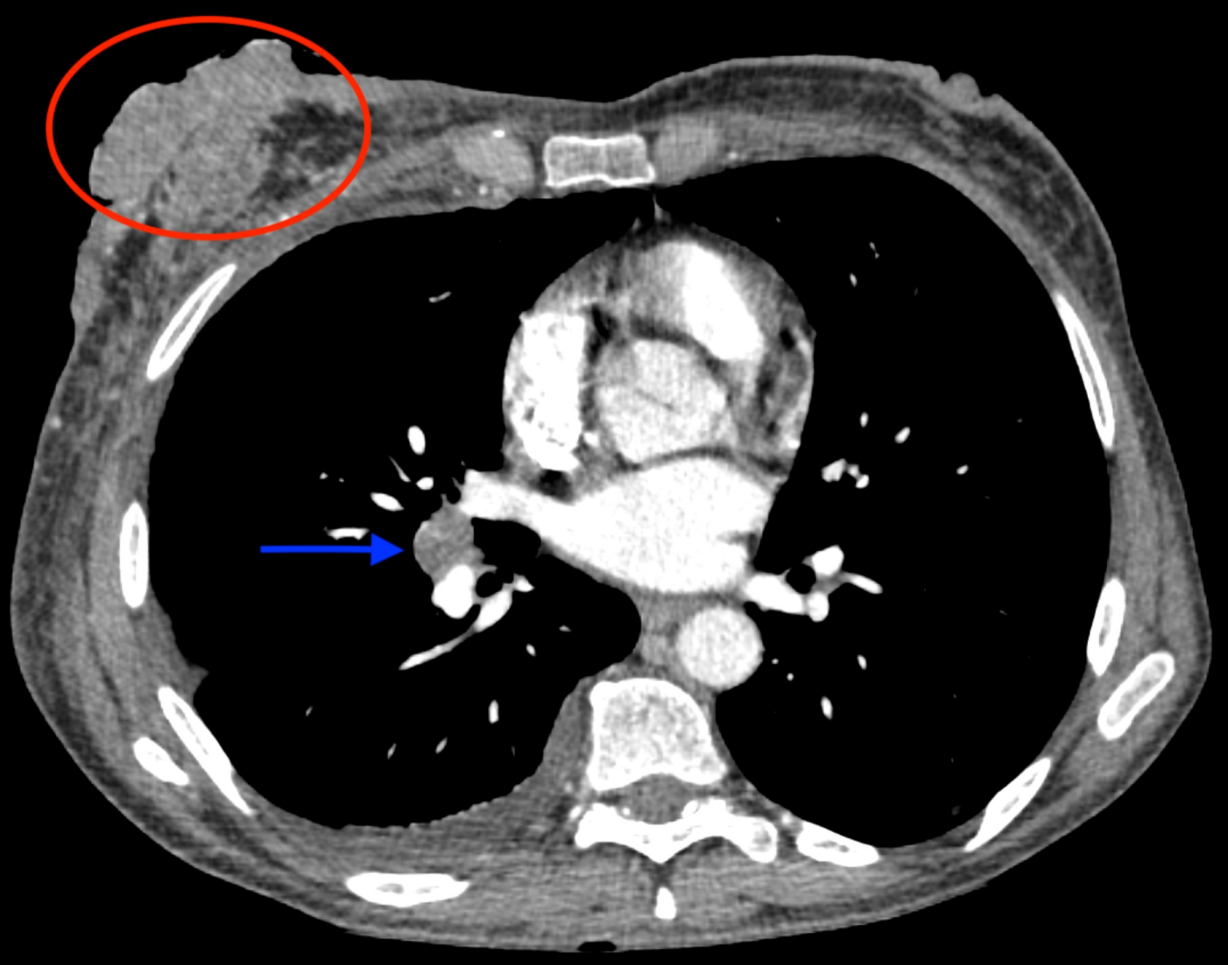
Axial image of the CT scan of chest at the level of nipples shows a large right breast mass (red circle) and right hilar lymphadenopathy (blue arrow)

**Figure 2:**
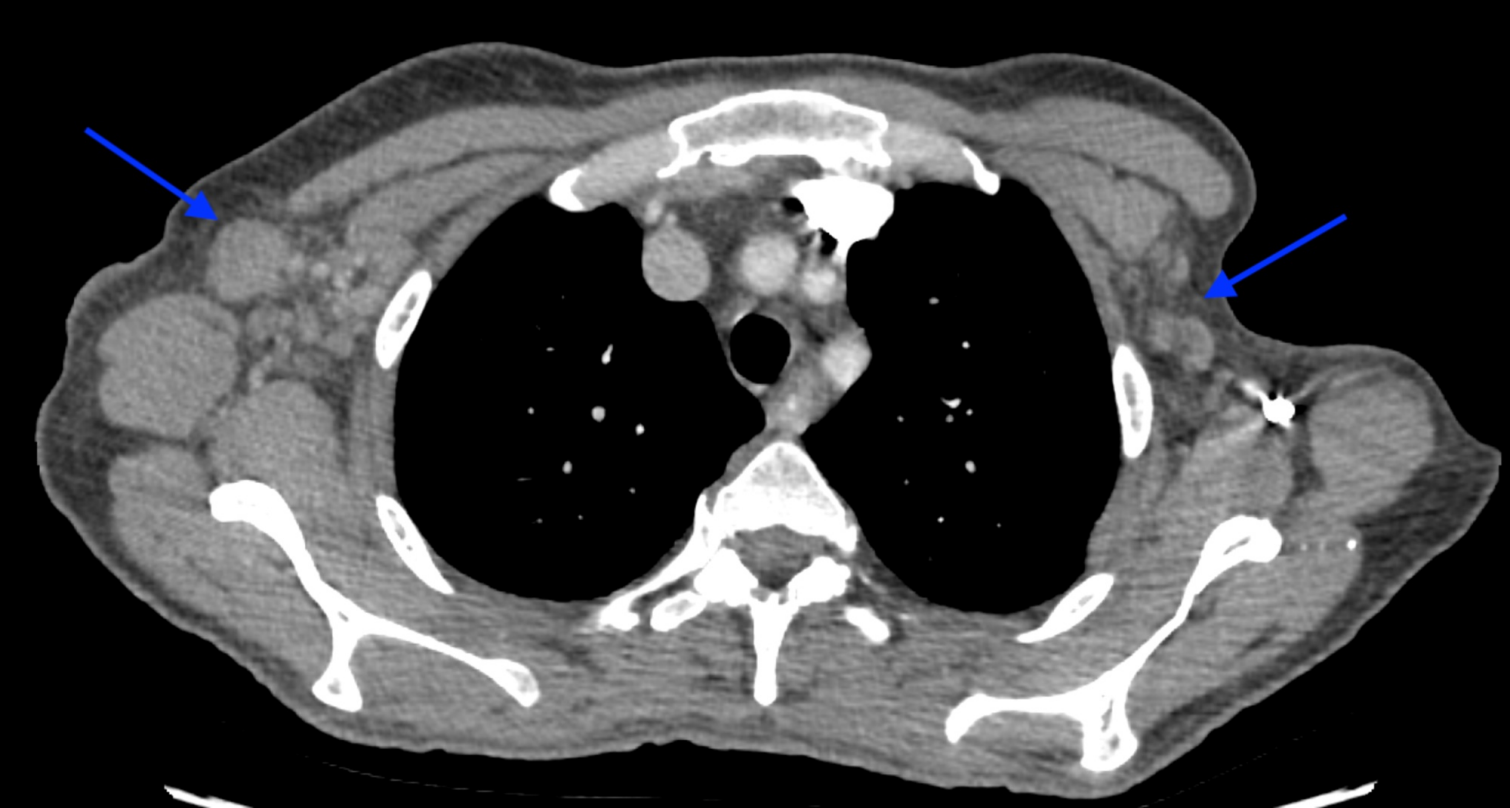
Axial image of the CT scan of chest at the level of axilla shows bilateral axillary lymphadenopathy (blue arrows)

**Figure 3:**
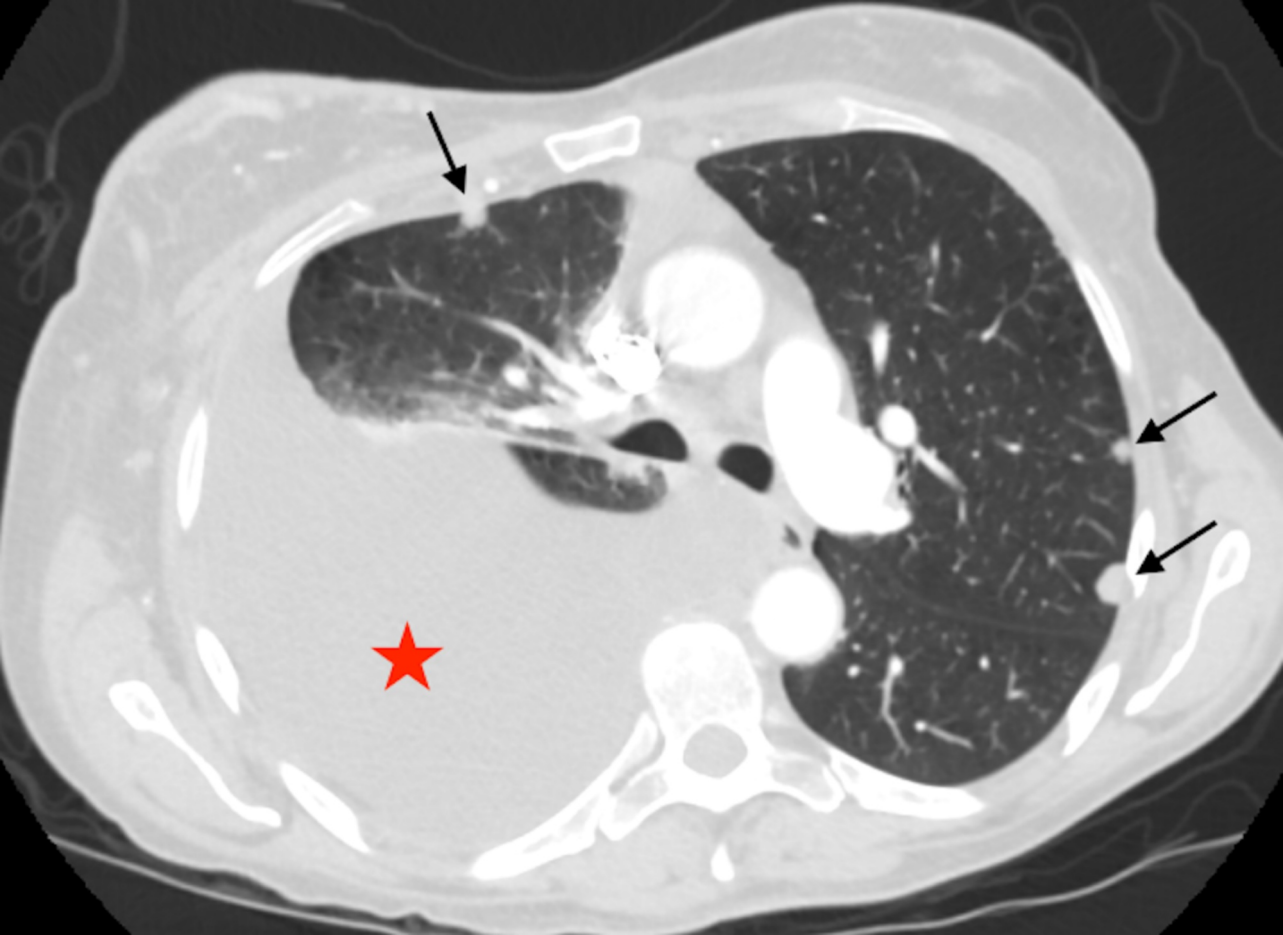
Lung window of an axial image of the CT scan of chest shows bilateral lung nodules (black arrows) and pleural effusion (red star)

A 49-year-old female presented to the hospital in July 2019, reporting a two-year history of a growing palpable right breast mass and nipple discharge. She explained that she did not seek medical attention sooner because of issues with her health insurance coverage. Pertinent positives included smoking history for past 13 years, intravenous drug use, family history of father passing away secondary to lung cancer, and mother passing away secondary to breast cancer. A right breast fungating mass was noted on physical exam. A thorough workup included fine needle aspiration (FNA) of right breast mass, culture of nipple discharge and CT of the chest, abdomen and pelvis. FNA results showed infiltrative ductal carcinoma, estrogen receptor negative, progesterone receptor negative, and human epidermal growth factor receptor 2 positive. Culture was positive for Staphylococcus and Pseudomonas. The infection responded to antibiotic treatment. Imaging demonstrated large right breast mass with satellite lesions, multistation lymphadenopathy, bilateral lung nodules, and pleural effusion (Figures 1-3). 

**Figure 4:**
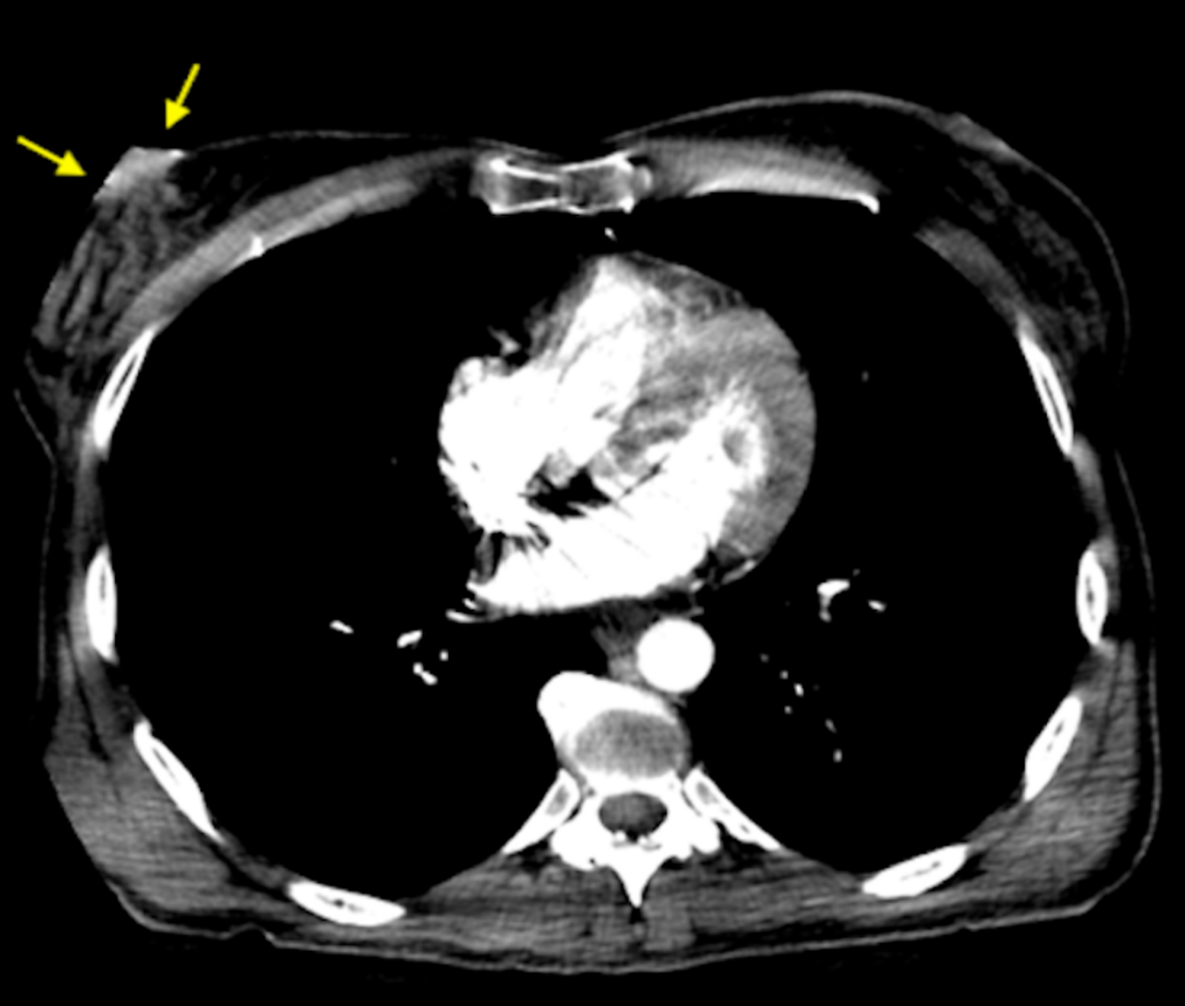
Axial image of a trauma protocol CT chest performed three years prior, in retrospect, shows asymmetric right nipple enhancement (yellow arrows), relative to the left nipple

Retrospectively, asymmetric right nipple enhancement was seen on a CT angiography of the chest performed as workup of motor vehicle collision, three years prior to current presentation (Figure 4).

The patient is currently undergoing chemotherapy and radiation treatment for stage IV metastatic breast cancer. This case illustrates that the breast and, particularly, the NAC can be a blind spot while reading CT chest for indications unrelated to the breast.

## Discussion

At CT, invasive ductal carcinoma can appear as a dense, spiculated mass with marked early and/or peripheral enhancement. Radiologists should characterize breast lesions as benign, indeterminate, or suspicious [4]. Irregular and spiculated margins and rim enhancement are the morphological features most predictive of malignancy. Invasive ductal carcinoma is the most common type of breast cancer detected as an incidental breast lesion on CT [5]. For incidentally detected indeterminate and suspicious breast lesions, additional workup is recommended.

Differential diagnosis for abnormal NAC enhancement includes dermatologic conditions like eczema, psoriasis; benign breast diseases like mastitis, breast abscess, papilloma, adenoma, leiomyoma; and malignant conditions like Paget disease and invasive ductal cancer [6-8]. Clinical history and physical examination are helpful for diagnosis in many cases. Additional imaging evaluations include standard dual-view mammography. When routine mammographic findings are negative, ultrasound and MRI can help facilitate the diagnosis.

The current guideline for asymmetric NAC enhancement on MRI is a referral to breast surgeon for appropriate clinical exam. If there is a clinically detected abnormality, punch biopsy is the next step. However, if there is no clinically detectable abnormality, a full diagnostic workup including diagnostic mammogram and ultrasound should be considered.

Dynamic contrast-enhanced MRI is the most sensitive test to evaluate the NAC and its enhancement patterns; it is especially useful in screening high-risk patients, for diagnostic workup of biopsy-proven breast cancer, and for treatment follow-up, as well as for unilateral spontaneous clear or bloody nipple discharge [9]. Physiologic NAC enhancement is either absent or described as a 1-2 mm zone of superficial linear dermal enhancement overlying a non-enhancing zone (NEZ) deep to the dermis with patchy or linear internal enhancement below the NEZ. Physiologic NAC enhancement when present is symmetric and shows persistent kinetics. Physiologic periareolar skin thickening is rare, and when present is smooth, bilateral, and symmetric.

Pathologic NAC should be suspected if any of the following MRI findings are noted: new changes in NAC morphology, new nipple flattening or inversion, asymmetric NAC enhancement, nodular or irregular internal enhancement below the NEZ, asymmetric or irregular periareolar skin thickening, and/or presence of a subareolar mass.

Recommendations for workup of asymmetric NAC enhancement detected by CT, as in our case, can be better ascertained with a larger case series in the future. However, we propose a similar approach to that described for NAC enhancement detected by MRI. Initial referral to breast surgeon for thorough history and clinical exam, followed by punch biopsy for any clinically detected abnormality, is appropriate. In the absence of a clinical abnormality, a diagnostic mammogram and ultrasound are performed, followed by ultrasound-guided biopsy of any detectable imaging abnormality. 

## Conclusions

The NAC remains an often-neglected area, not only on CT scans done for non-breast symptoms, but also occasionally on screening mammograms. This report outlines how the role of radiologists includes, detecting and characterizing incidentally imaged breast lesions on CT scans and recommending further workup if indicated. 

Abnormal NAC enhancement on CT or MRI can be a sign of breast malignancy, and hence requires a focused approach, multidisciplinary team effort including considering surgical consultation, patient-centered biopsy planning, and careful follow-up.
